# MdMYB41L mediates stamen development and anthocyanin biosynthesis in apple

**DOI:** 10.3389/fpls.2026.1905435

**Published:** 2026-07-30

**Authors:** Kuo Yang, Ruihong Sun, Yanfu Pan, Zhudong Xing, Yanyi Zhang, Hengjia Zhang, Chongyang Li

**Affiliations:** College of Agriculture and Biology, Liaocheng University, Liaocheng, China

**Keywords:** anthocyanin, apple, MdMYB41L, seed development, stamen developmental

## Abstract

MYB proteins play crucial roles in plant growth and development. In this study, we characterized MdMYB41L, a MYB transcription factor from apple (*Malus domestica*). Expression analysis revealed that *MdMYB41L* was highly expressed in leaves and anthers, and the protein was exclusively localized to the nucleus. Overexpression of *MdMYB41L* in *Arabidopsis* caused shorter siliques, anther indehiscence, and a lower seed setting rate. Artificial pollination analysis demonstrated that the stamen developmental defect resulting from *MdMYB41L* overexpression led to decreased fertility. The seeds from transgenic lines showed a dramatic increase in length, width, area, and weight. Overexpression of *MdMYB41L* suppresses anthocyanin accumulation through downregulating the expression of anthocyanin biosynthesis‐related genes. Collectively, our findings highlight the important role of *MdMYB41L* in regulating stamen development and anthocyanin biosynthesis.

## 1 Introduction

In seed plants, upon deposition on the stigma, pollen grains undergo adhesion, hydration, and germination, followed by growth through the style prior to fertilization. Anthers are key components of stamens. Anther dehiscence and filament elongation represent the terminal phases of anther development (Zúñiga-Mayo et al., 2023). During the mature pollen stage, the septum degenerates, causing the anther to become bilocular, and the stomium subsequently ruptures to facilitate pollen release (Wilson et al., 2011). If any physiological process of stamen elongation, pollen maturation, or anther dehiscence is disrupted, it can lead to male sterility in plants (Dai et al., 2019; Åstrand et al., 2021; He et al., 2023). Numerous genes have been demonstrated to participate in the regulation of stamen development (Marciniak and Przedniczek, 2019; Su et al., 2023; Sui et al., 2023; Wiese et al., 2024; Li et al., 2025).

MYB transcription factors (TFs) constitute one of the largest transcription factor families in plants, with members distributed across diverse species. MYB proteins are characterized by a conserved MYB domain, among which the R2R3-MYB type represents the most extensively studied subfamily (Wu et al., 2024). MYB TFs are extensively involved in various biological processes, including plant growth, development, stress responses, and secondary metabolism (Dubos et al., 2010; Wu et al., 2025; Zhang et al., 2025). Notably, MYB proteins have been demonstrated to play critical roles in plant fertility. In *Arabidopsis*, *myb108 myb24* double mutant exhibits reduced pollen tube elongation, accompanied by lower pollen viability and germination, leading to decreased fertility (Mandaokar and Browse, 2009). MYB transcription factors regulate anther development in plants. In pepper, silencing of *CaMYB108* led to delayed anther dehiscence and decreased pollen viability (Sun et al., 2019). The *myb21 myb24* double mutant of *Arabidopsis* displays abnormalities in pollen maturation, anther dehiscence, and filament elongation, which collectively cause male sterility (Song et al., 2011). MYB21 and MYB24 participate in jasmonate-mediated stamen development through the bHLH-MYB complexes (Chen et al., 2016; Huang et al., 2017). Accordingly, it is essential to identify MYB proteins implicated in stamen development. In addition to their functions in reproductive development, MYB proteins are also key regulators of plant secondary metabolism. Anthocyanin biosynthesis is a major branch of the phenylpropanoid pathway, which is catalyzed by enzymes encoded by structural genes such as *CHS*, *CHI*, *F3H*, *DFR*, *ANS*, and *UFGT* (Kuang et al., 2025). MYB proteins often assemble with bHLH and WD40 proteins into the MBW complex, which synergistically activates or represses the expression of structural genes, thereby fine-tuning anthocyanin accumulation (Liu et al., 2021; Muhammad et al., 2024; Sharma et al., 2024; Zhao et al., 2025; He et al., 2026). The MYB protein FaMYB5 positively regulates anthocyanin and proanthocyanidin biosynthesis by directly activating the transcription of *F3’H* and *LAR* in strawberry. FaMYB5 forms an MBW complex with FaEGL3 and FaLWD1/FaLWD1-like to modulate flavonoid metabolism (Jiang et al., 2023). Under low nitrate conditions, MsMYB62-like suppresses the expression of *MsF3’H*, thereby negatively regulating anthocyanin biosynthesis in *Malus spectabilis* (Meng et al., 2023). In apple, MdMYB73 negatively regulates anthocyanin biosynthesis through suppressing *MdUFGT*. Furthermore, MdMYB73 competes with MdMYB1, a positive regulator of anthocyanin biosynthesis, for binding to the *MdUFGT* promoter, thus exerting an inhibitory effect on anthocyanin accumulation (Zhang et al., 2026).

Apple is rich in mineral elements, vitamins, and anthocyanins, which make it popular among consumers. Here, we isolated and characterized *MdMYB41L*, an MYB gene from apple. We found that *MdMYB41L* was highly expressed in anthers. *MdMYB41L*-overexpressing *Arabidopsis* exhibited significantly reduced fertility and produced larger seeds. Artificial pollination experiments confirmed that impaired pollination was the primary cause of the seed phenotype observed in these transgenic lines. Furthermore, overexpression of *MdMYB41L* in apple calli decreased anthocyanin accumulation and suppressed the expression of anthocyanin biosynthetic genes. Collectively, our findings demonstrate that *MdMYB41L* regulates stamen development and anthocyanin biosynthesis.

## 2 Materials and Methods

### 2.1 Plant materials and growth conditions

*Arabidopsis thaliana* plants were grown in the greenhouse under long day conditions (16 h light/8 h dark) at 22°C. The Columbia‐0 ecotype was used as the wild-type *Arabidopsis*. *Arabidopsis* seeds were surface-sterilized with 75% ethanol and 2.6% sodium hypochlorite, placed at 4 °C for 3 days. They were then sown on 1/2MS medium. The apple calli ‘Orin’ were cultured on MS medium supplemented with 1.5 mg/L 2,4-D and 0.4 mg/L 6-BA at 22 °C in the dark, and subcultured every two weeks (Yang et al., 2021a).

### 2.2 Vector construction and plant transformation

The full-length cDNA of *MdMYB41L* was inserted into the pRI-101 plasmid to generate the overexpression vector. The *MdMYB41L* overexpression vector was transformed into *Agrobacterium tumefaciens* strain EHA105, which was then introduced into apple calli (Yang et al., 2021b). In order to generate transgenic lines, we transferred overexpression vector into *Agrobacterium tumefaciens* strain GV3101 and used an *Agrobacterium-*mediated method to transform *Arabidopsis*. All phenotypic analyses of the transgenic *Arabidopsis* were conducted using homozygous T3 generation lines. The primers used in the study are listed in **Supplementary Table 1**.

### 2.3 Seed size measurement.

We observed and captured images of mature dry seeds under a stereomicroscope (SZX16, Olympus). The ImageJ 1.54g software was used to measure seed length, width, and area. *Arabidopsis* seeds were weighed by analytical microbalance.

### 2.4 Differential interference contrast (DIC) microscopy

Immature seeds extracted from siliques were fixed at 4°C in Hoyer’s solution (chloral hydrate : glycerol : water = 8 : 1 : 3) for DIC observations. A Nicon digital sight 10 cameras were used to capture images. ImageJ software was used to measure ovule size and embryo size from the DIC images.

### 2.5 Gene expression analysis

Total RNA was extracted using RNA plant extraction kits (Tiangen, Beijing, China). The cDNA was synthesized by reverse transcription kit (TaKaRa, Dalian, China). Quantitative real-time PCR (qRT-PCR) was performed on a real-time PCR system using SYBR Green reagents (Vazyme, Nanjing, China) with cDNA as the template. The *MdActin* was used as an internal control. Relative expression levels were calculated using the 2^−ΔΔCt^ method (Wang et al., 2020; Sui et al., 2023).

### 2.6 Subcellular localization analysis

The full-length CDS of *MdMYB41L* was fused into the pPZP211-GFP vector to obtain *35S::MdMYB41L-GFP*. The recombinant plasmid was introduced into *A. tumefaciens* strain GV3101. Then, they were injected into *N. benthamiana* leaves and incubated in the dark for 3 days. Fluorescence signals were captured using a confocal laser‐scanning microscope (FV1200, Olympus, Japan).

### 2.7 GUS staining

The 1592 bp promoter fragment of *MdMYB41L* was inserted into the *pCAMBIA1391* vector to obtain the *ProMdMYB41L::GUS*. The recombinant plasmid was transformed into *Arabidopsis*. Leaves, stems, roots, and flowers were stained using a GUS staining kit (Solarbio, Beijing, China) according to the manufacturer’s instructions (Yang et al., 2021b).

### 2.8 Determination of anthocyanin contents

Total anthocyanins were extracted using the ethanol-HCl method. Apple calli were immersed in an extraction buffer (95% anhydrous ethanol mixed with 1.5 M HCl) at room temperature for 24 h. The absorbance of the extract was measured at 530, 620, and 650 nm using a spectrophotometer, and the anthocyanin content was calculated according to described methods (An et al., 2018).

## 3 Results

### 3.1 Bioinformatics characterization of the MdMYB41L in apple

MYB TFs are known to be extensively involved in plant development. Here, we isolated a MYB gene, designated *MdMYB41L*, from apple. *MdMYB41L* contains a 1077 bp open reading frame and encodes a protein of 358 amino acids. In addition, MdMYB41L has a theoretical pI of 6.37, a molecular weight of 39,675.76 Da, and a grand average of hydropathicity (GRAVY) of -0.488. Sequence alignment revealed that MdMYB41L protein shares 96.09% sequence identity with a MYB protein from *Pyrus bretschneideri*. We further compared the amino acid sequences of MdMYB41L with those from six other plant species. Consistent with other MYB proteins, MdMYB41L possesses two conserved MYB domains at its N-terminus (**Figure 1**).

**Figure 1:**
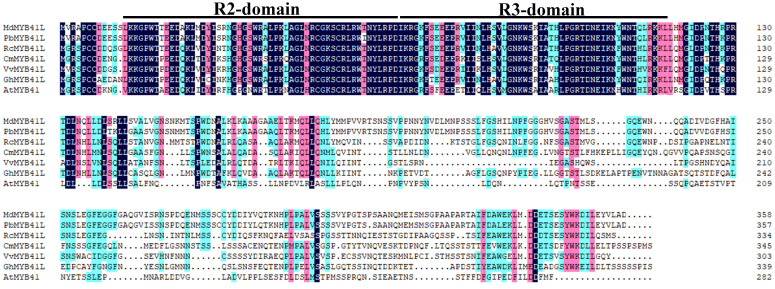
Amino acid sequence alignment of MYB proteins. The two conserved MYB domains (R2-domain and R3-domain) of MYB proteins are marked with black lines. MdMYB41L: MD06G1037300 *Malus domestica*; PbMYB41L: XP_009371224.2 *Pyrus bretschneideri*; RcMYB41L: XP_024173796.1 *Rosa chinensis*; CmMYB41L: KAF3964320.1 *Castanea mollissima*; VvMYB41L: CBI33861.3 *Vitis vinifera*; GhMYB41L: XP_016721289.1 *Gossypium hirsutum*; AtMYB41L: AT4G28110 *Arabidopsis thaliana*.

### 3.2 Expression pattern analysis and subcellular localization of MdMYB41L

MYB proteins are widely distributed across plant species (Li et al., 2019; Wang et al., 2021). We explored the tissue expression pattern of *MdMYB41L* through GUS staining. The promoter fragment of *MdMYB41L* was fused into the *pCAMBIA1300::GUS* vector and transformed into *Arabidopsis* to obtain *ProMdMYB41L::GUS* transgenic *Arabidopsis.* As shown in **Figure 2A**, the GUS staining displayed that *MdMYB41L* was expressed in roots, stems, leaves, and flowers, with the highest expression in leaves and anthers. To determine the subcellular localization of MdMYB41L, we generated *35S::MdMYB41L-GFP* recombinant plasmid. The recombinant construct was introduced into *A. tumefaciens* strain GV3101, and then was injected into tobacco leaves. After 3 days, the GFP fluorescence of MdMYB41L was detected using a confocal laser‐scanning microscope. As shown in **Figure 2B**, MdMYB41L was specifically localized in the nucleus.

**Figure 2:**
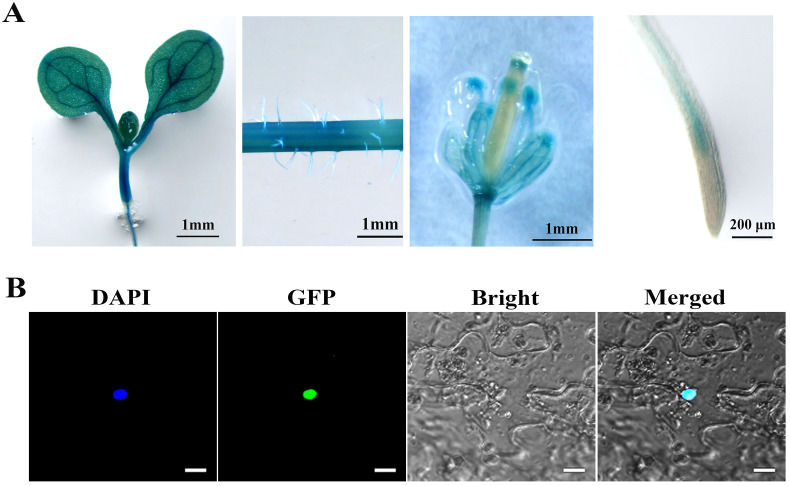
Expression pattern and subcellular localization of MdMYB41L. **(A)** GUS staining of *ProMdMYB41L::GUS* transgenic *Arabidopsis* in roots, stems, leaves and flowers. **(B)** Subcellular localization of MdMYB41L protein. DAPI dye specific staining of cell nuclei. Bright represents a bright field. Scale bar: 10 μm.

### 3.3 Overexpression of *MdMYB41L* alters silique development

To investigate the function of *MdMYB41L*, we transformed *MdMYB41L* into *Arabidopsis.* As a result, three stable overexpression lines (OE1, OE2, and OE3) were generated. The *MdMYB41L-OE* lines were confirmed at the transcriptional and DNA levels (**Figures 3A, B**). Through phenotype analysis, we found no prominent difference in plant height between wild-type (WT) and *MdMYB41L-OE* lines under normal growth conditions. However, we found that siliques development in the *MdMYB41L-OE* lines was defective, with many aborted siliques and significantly shorter siliques than those of WT (**Figures 3C–E**).

**Figure 3:**
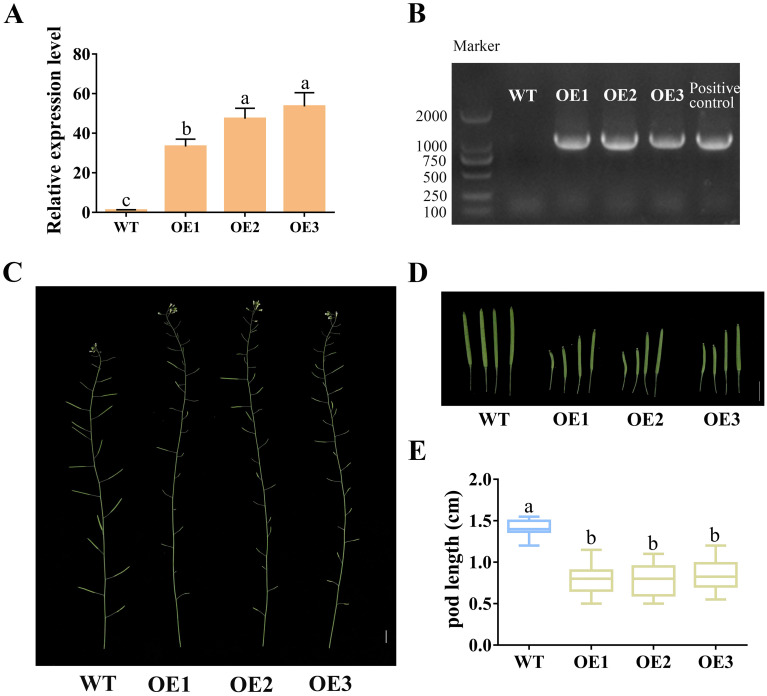
*MdMYB41L* affects siliques development. **(A)** qRT-PCR detected the expression level of *MdMYB41L* in WT and *MdMYB41L-OE.* **(B)** The *MdMYB41L-OE* lines were identified through PCR amplification. **(C)** Main inflorescences of WT and *MdMYB41L-OE* lines. Bars: 1 cm. **(D, E)** Phenotype and length of siliques in WT and *MdMYB41L-OE.* Bars: 0.5 cm. For **(E)** error bars indicate SD (n=40). different lowercase letters represent significant differences by one-way ANOVA, followed by Tukey’s test (*p* < 0.05). All data were obtained from three independent biological replicates.

### 3.4 Overexpression of *MdMYB41L* reduces the fertility in *Arabidopsis*

We observed that, within the same inflorescence, most flowers either failed to set seeds or set only a few, whereas a small proportion developed normally into siliques. Furthermore, *MdMYB41L-OE* lines exhibited significant reduced fertility compared with WT (**Figure 4A**). The silique seed set rate in *MdMYB41L-OE* lines was notably lower than that of WT (**Figure 4B**). In addition, seed yield per plant was markedly decreased in the OE lines compared to WT (**Figure 4C**). These results indicate that *MdMYB41L* negatively regulates fertility in *Arabidopsis*.

**Figure 4:**
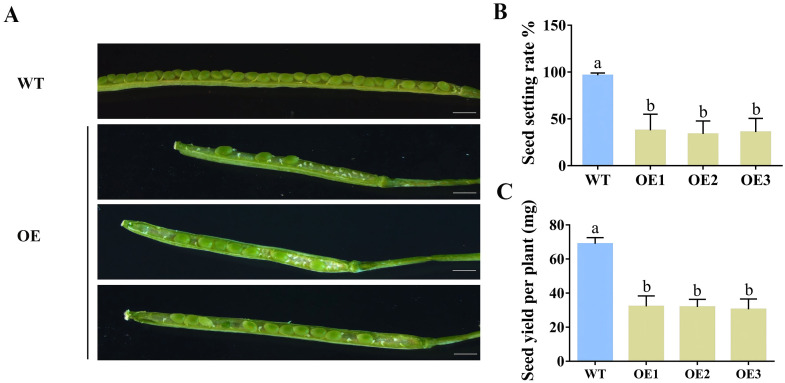
Overexpression of *MdMYB41L* reduces fertility in *Arabidopsis*. **(A)** Dissected silique phenotypes of *Arabidopsis*. Bar: 1 mm. **(B)** Seed setting rate in WT and *MdMYB41L-OE* lines (n=100). **(C)** Seed yield per plant of WT and *MdMYB41L-OE* lines (n=30). For B, C, different lowercase letters represent significant differences by one-way ANOVA, followed by Tukey’s test (*p* < 0.05). All data were obtained from three independent biological replicates.

To investigate the mechanism by which *MdMYB41L* affects fertility, we observed the anther morphology of WT and *MdMYB41L-OE*. As shown in **Figures 5**, *MdMYB41L-OE* flowers exhibited anther indehiscence, with only a minority of anthers showing normal dehiscence (**Figures 5A, B**). Subsequently, cross-pollination assays were conducted between WT and the *MdMYB41L-OE1* line. When *MdMYB41L-OE1* was used as the female parent and WT as the male parent, the silique seed set rate showed no notable difference compared with that of WT, indicating that female gametophyte function in *MdMYB41L-OE1* remained normal (**Figure 6**). These results suggest that the reduced fertility of *MdMYB41L-OE* is largely attributable to defective anther dehiscence.

**Figure 5:**
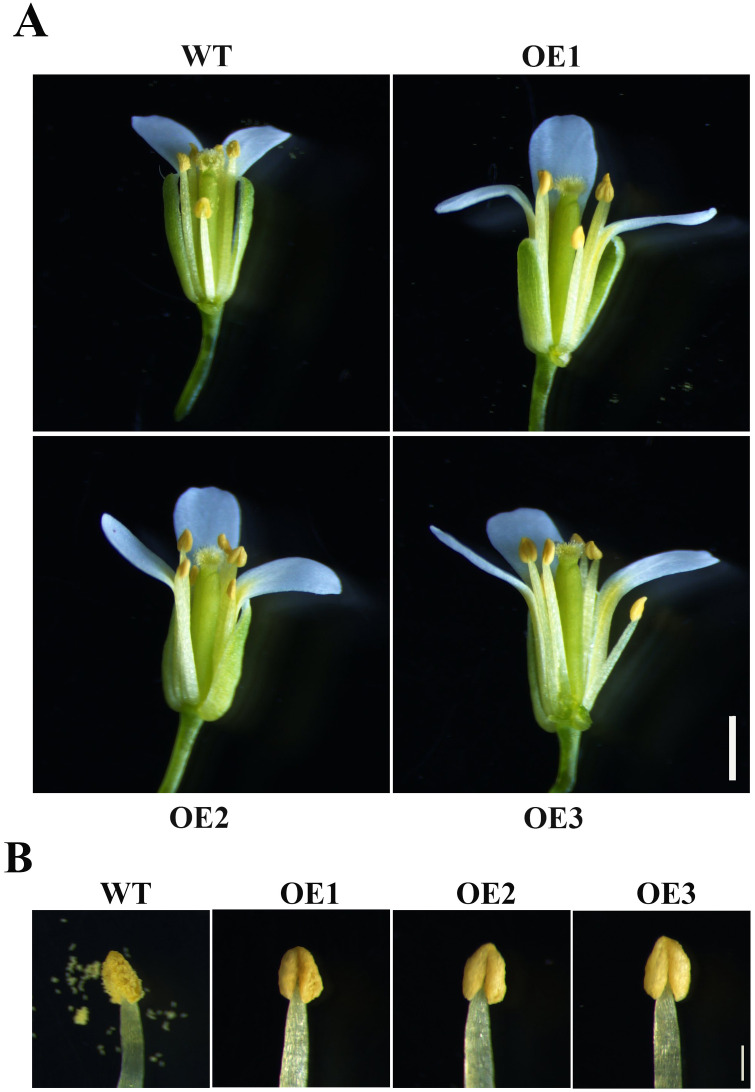
Overexpression of *MdMYB41L* affects the stamens development. **(A)**. Comparison of flowers. Bar: 1 mm. **(B)** Comparison of anther in WT and *MdMYB41L-OE* plants. WT showed obvious anther dehiscence. Bar: 200 μm.

**Figure 6:**
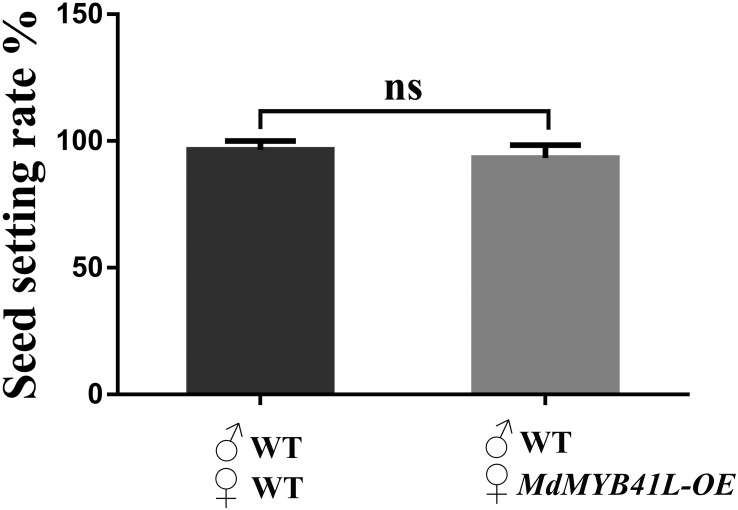
Crosses between the WT and the *MdMYB41L-OE1* plants. The siliques are collected at 15 days after pollination. Statistical analysis of silique seed set. Student’s *t* test was performed (*p* < 0.05; n=20). ns, not significant.

### 3.5 Overexpression of *MdMYB41L* increases the seed size

MYB proteins function as established regulators of seed size in plants (Zhang et al., 2023; Wang et al., 2024). In this study, we found that overexpression of *MdMYB41L* not only led to decreased fertility, but also exhibited significantly enlarged seeds and mature embryos (**Figures 7A, B**). Statistical analysis showed that the length, width, area, and weight of seeds of *MdMYB41L-OE* lines were dramatically greater than those of WT (**Figures 7D–G**). Similarly, we observed that *MdMYB41L*-*OE* cotyledons were markedly larger than those of WT at 9 days post-germination (**Figures 7C, H**).

**Figure 7:**
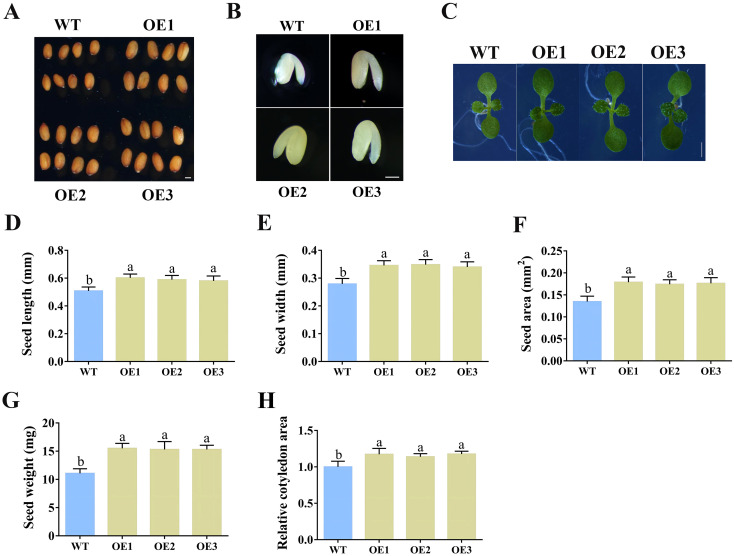
*MdMYB41L* affects seed size in *Arabidopsis*. **(A)** Seed phenotype of WT and *MdMYB41L-OE* lines. Bar=0.2 mm. **(B)** Mature embryo phenotype of WT and *MdMYB41L-OE* lines. Bar=0.2 mm. **(C)** The 9-d-old seedlings of WT and *MdMYB41L-OE* lines. Bar=0.2 mm. **(D–F)** the length **(D)**, width **(E)**, area **(F)** of WT and *MdMYB41L-OE* lines (n=200). **(G)** 500-seed weight of WT and *MdMYB41L-OE* lines (n=3). **(H)** Relative cotyledon area of WT and *MdMYB41L-OE* lines (n=20). The WT was set to 1. For **(B–H)**, different lowercase letters represent significant differences by one-way ANOVA, followed by Tukey’s test (*p* < 0.05). All data were obtained from three independent biological replicates.

### 3.6 Overexpression of *MdMYB41L* affects the seed development

To elucidate the role of *MdMYB41L* in seed development, we performed cytological analyses on seeds collected at different days after pollination (DAP). Seeds at 3, 6, and 8 DAP were isolated from the siliques of WT and *MdMYB41L-OE1*, and were fixed in Hoyer’s solution. As shown in **Figure 8**, embryo development proceeded similarly in WT and *MdMYB41L-OE1* from 3 to 8 DAP. After the globular stage (3 DAP), their differences in seed cavity became increasingly pronounced (**Figure 8A**). At both the torpedo and bent cotyledon stages, the embryo and ovule sizes in *MdMYB41L-OE1* were significantly greater than those in WT (**Figures 8B, C**). Accordingly, these results suggest that the increased seed size of *MdMYB41L-OE* lines is caused by embryo enlargement.

**Figure 8:**
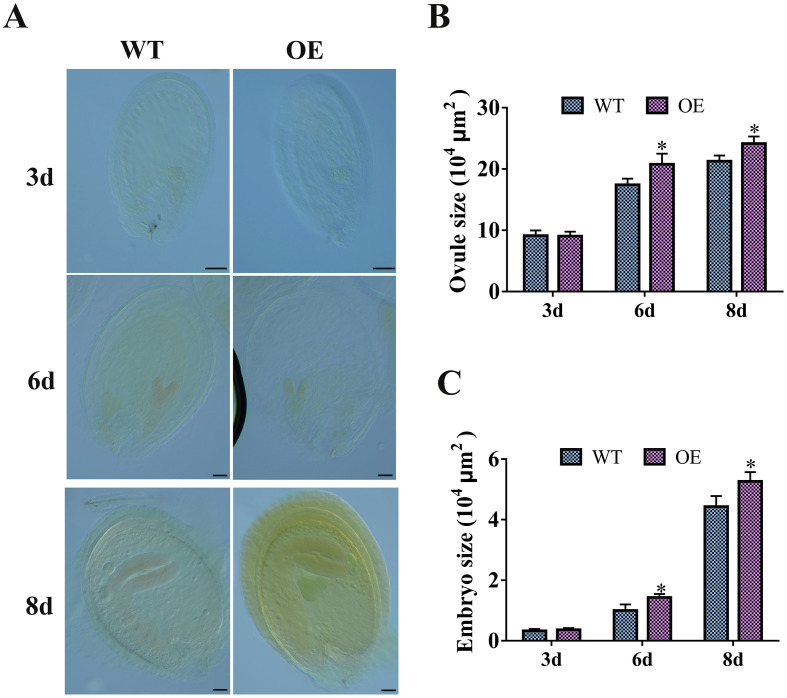
Seed development of WT and *MdMYB41L-OE*. **(A)** DIC microscopy of WT and *MdMYB41L-OE* lines from 3 DAP, 6 DAP, and 8 DAP. Scale bars: 50 µm. **(B, C)** The ovule size **(B)** and embryo size **(C)** of WT and *MdMYB41L-OE*. Asterisks represent significant differences (*p<* 0.05, Student’s *t* test).

### 3.7 MdMYB41L negatively regulates anthocyanin accumulation

MYB proteins play critical roles in regulating anthocyanin biosynthesis across various plant species (Yang et al., 2022). To investigate the function of *MdMYB41L* in anthocyanin biosynthesis, we generated two *MdMYB41L*-overexpressing apple calli lines (OE1 and OE2) (**Figure 9B**). Compared with WT, both *MdMYB41L-OE* transgenic calli lines exhibited lower anthocyanin content (**Figures 9A, C**). Consistent with anthocyanin phenotype, qRT-PCR analysis revealed that the transcript levels of anthocyanin biosynthesis-related genes *MdDFR*, *MdANS*, and *MdF3H* were significantly downregulated in *MdMYB41L-OE* calli (**Figure 9D**). Collectively, these results demonstrate that *MdMYB41L* functions as a negative regulator of anthocyanin accumulation in apple.

**Figure 9:**
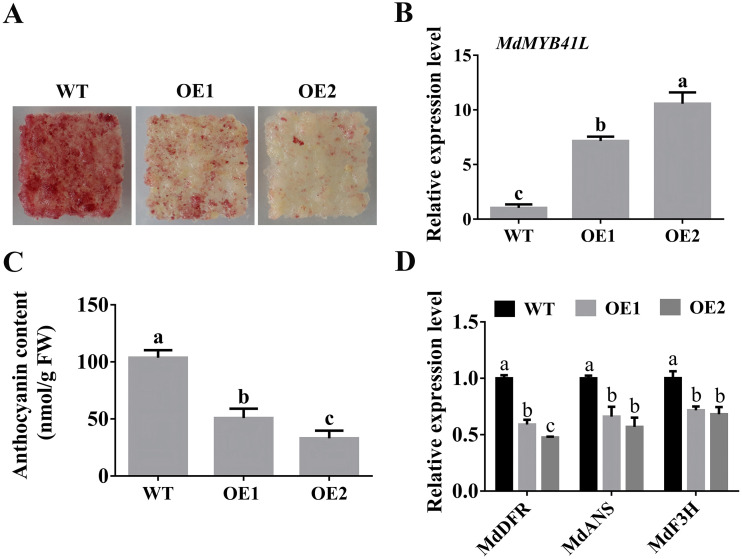
MdMYB41L inhibits anthocyanin accumulation. **(A)** Phenotype of anthocyanin accumulation. The 15-day-old WT and *MdMYB41L-OE* apple calli were maintained in a plant greenhouse at 22 °C under light conditions (photon flux density of approximately 70 μmol·m^−2^·s^−1^) for 10 days. **(B)** The expression of *MdMYB41L* in apple calli. **(C)** The anthocyanin content. **(D)** The expression of anthocyanin biosynthesis‐related genes (*MdDFR*, *MdANS*, and *MdF3H*). *MdActin* acted as the reference. For **B-D**, different lowercase letters represent significant differences by one-way ANOVA, followed by Tukey’s test (*p* < 0.05). All data were obtained from three independent biological replicates.

## 4 Discussion

The normal dehiscence of anthers and the timely release of pollen are closely related to plants fertility (Åstrand et al., 2021). MYB proteins are involved in plant reproductive processes, such as anther development, anther dehiscence, and tapetum development (Zhang et al., 2025). Here, we identified an R2R3 MYB gene, *MdMYB41L*, from apple. We demonstrated that overexpression of *MdMYB41L* led to anther indehiscence in *Arabidopsis* (**Figure 5**), thereby reducing the seed setting rate. Sorbitol participates in the processes of stamen development, pollen germination, and pollen tube growth through the apple R2R3 MYB transcription factor MdMYB39L (Meng et al., 2018; Li et al., 2020). Given the close phylogenetic relationship between MdMYB41L and MdMYB39L, MdMYB41L may exert similar functions in reproductive development.

Overexpression of *MdMYB41L* in *Arabidopsis* resulted in a similar number of ovules as in WT; however, not all ovules developed into seeds, leading to a significant reduction in fertility in the *MdMYB41L* transgenic *Arabidopsis*. Artificial crossing experiments showed that female gametophyte function was normal in transgenic *Arabidopsis* (**Figure 6**). Taken together with the observed anther indehiscence phenotype, these findings demonstrate that the reduced fertility of *MdMYB41L-OE* lines is primarily attributable to defect in stamen development.

The *Arabidopsis* MYB26 regulates secondary thickening by altering the expression of *NST1* and *NST2*, and its overexpression causes ectopic secondary thickening and anther dehiscence failure (Yang et al., 2007). Similarly, ectopic expression of the *Brassica campestris* R2R3-MYB gene BcMF28 in *Arabidopsis* leads to shortened filaments, indehiscent anthers, and aborted pollen, due to the non-degeneration of the septum and stomium during late anther development (Shen et al., 2019). In eggplant, overexpression of *SmMYB108* promotes early anther dehiscence. The anther development-related genes (*SmMYB21*, *SmARF6*, and *SmARF8*) directly activate *SmMYB108* transcription (Hu et al., 2023). The specific pathway through which MdMYB41L regulates anther development requires further investigation.

Interestingly, *MdMYB41L-OE Arabidopsis* seeds were larger than those of the WT (**Figure 7**). Seed size is determined by the coordinated development of the seed coat, endosperm, and embryo, as well as on genetic, environmental, and physiological factors (Doughty et al., 2014; Gnan et al., 2014; Orozco-Arroyo et al., 2015; Zhang et al., 2023). Given that artificial cross-pollination of *MdMYB41L*-*OE* plants restored the WT seed size phenotype, the increased seed size in *MdMYB41L*-*OE* transgenic lines is likely due to reduced seed set. In *MdMYB41L-OE* plants, nutrients allocated to flowers are distributed among fewer developing seeds, which may consequently accumulate more storage compounds (Herridge et al., 2011; Rolletschek et al., 2021; Wang et al., 2023; Li et al., 2026). Similar observations have been reported in other genes and species (Vergès et al., 2021). Nevertheless, an alternative possibility cannot be excluded. Numerous studies have demonstrated that MYB transcription factors can directly regulate seed size (Zhang et al., 2025). Therefore, it is plausible that MdMYB41L may directly influence seed development, although this hypothesis requires further validation.

MYB TFs play indispensable roles in plant secondary metabolism (Zhang et al., 2025). Overexpression of *MdMYB41L* reduced anthocyanin accumulation in apple calli. Consistently, qRT-PCR analysis revealed that the transcript levels of *MdDFR*, *MdF3H*, and *MdANS* were downregulated in *MdMYB41L-OE* apple calli (**Figure 9**), suggesting that MdMYB41L may suppress anthocyanin biosynthesis by suppressing the expression of anthocyanin synthesis related genes. MdMYB1 and its alleles act as positive regulators of anthocyanin biosynthesis by activating *MdDFR* and *MdUF3GT* to promote anthocyanin biosynthesis (Takos et al., 2006; An et al., 2020). MYB proteins that negatively regulate anthocyanin biosynthesis have also been identified in various plant species (Li et al., 2022). MdMYB6 inhibited anthocyanin accumulation and downregulated the expression of anthocyanin biosynthesis-related genes (Wang et al., 2018). Overexpression of *MdMYBL2* decreased anthocyanin accumulation in apple calli and inhibited the transcript levels of *MdDFR*, *MdUFGT*, *MdMYB10*, and *MdbHLH3* (Gao et al., 2011).

## 5 Conclusion

In this study, we identified and characterized *MdMYB41L*, an R2R3 MYB TF from apple. Overexpression of *MdMYB41L* leads to anther indehiscence, thereby reducing plant fertility. In addition, MdMYB41L negatively regulates anthocyanin accumulation in apple calli. Our findings offer novel insights into the regulatory mechanisms underlying MYB transcription factor-mediated stamen development and anthocyanin biosynthesis in plants and will contribute to future studies aimed at deciphering and utilizing this regulatory network.

## Data Availability

The original contributions presented in the study are included in the article/**Supplementary Material**. Further inquiries can be directed to the corresponding author/s.

## References

[B1] An J.-P. Wang X.-F. Hao Y.-J. (2020). BTB/TAZ protein MdBT2 integrates multiple hormonal and environmental signals to regulate anthocyanin biosynthesis in apple. J. Integr. Plant Biol. 62, 1643–1646. doi: 10.1111/jipb.12940 32298027

[B2] An J.-P. Yao J.-F. Xu R.-R. You C.-X. Wang X.-F. Hao Y.-J. (2018). Apple bZIP transcription factor MdbZIP44 regulates abscisic acid-promoted anthocyanin accumulation. Plant Cell Environ. 41, 2678–2692. doi: 10.1111/pce.13393 29940702

[B3] Åstrand J. Knight C. Robson J. Talle B. Wilson Z. A. (2021). Evolution and diversity of the angiosperm anther: trends in function and development. Plant Reprod. 34, 307–319. doi: 10.1007/s00497-021-00416-1 34173886 PMC8566645

[B4] Chen X. Huang H. Qi T. Liu B. Song S. (2016). New perspective of the bHLH-MYB complex in jasmonate-regulated plant fertility in arabidopsis. Plant Signal. Behav. 11, e1135280. doi: 10.1080/15592324.2015.1135280 26829586 PMC4883827

[B5] Dai S.-Y. Hsu W.-H. Yang C.-H. (2019). The gene ANTHER DEHISCENCE REPRESSOR (ADR) controls male fertility by suppressing the ROS accumulation and anther cell wall thickening in Arabidopsis. Sci. Rep. 9, 5112. doi: 10.1038/s41598-019-41382-z 30911018 PMC6434047

[B6] Doughty J. Aljabri M. Scott R. J. (2014). Flavonoids and the regulation of seed size in Arabidopsis. Biochem. Soc Trans. 42, 364–369. doi: 10.1042/bst20140040 24646245

[B7] Dubos C. Stracke R. Grotewold E. Weisshaar B. Martin C. Lepiniec L. (2010). MYB transcription factors in Arabidopsis. Trends Plant Sci. 15, 573–581. doi: 10.1016/j.tplants.2010.06.005 20674465

[B8] Gao J.-J. Shen X.-F. Zhang Z. Peng R.-H. Xiong A.-S. Xu J. . (2011). The myb transcription factor MdMYB6 suppresses anthocyanin biosynthesis in transgenic Arabidopsis. Plant Cell. Tissue Organ. Culture (PCTOC) 106, 235–242. doi: 10.1007/s11240-010-9912-4 30311153

[B9] Gnan S. Priest A. Kover P. X. (2014). The genetic basis of natural variation in seed size and seed number and their trade-off using Arabidopsis thaliana MAGIC lines. Genetics 198, 1751–1758. doi: 10.1534/genetics.114.170746 25313128 PMC4256784

[B10] He Y. He X. Wang X. Hao M. Gao J. Wang Y. . (2023). An EPFL peptide signaling pathway promotes stamen elongation via enhancing filament cell proliferation to ensure successful self-pollination in Arabidopsis thaliana. New Phytol. 238, 1045–1058. doi: 10.1111/nph.18806 36772858

[B11] He M. Li J. Jin W. Liu J. (2026). Unraveling the genetic and molecular mechanisms of anthocyanin biosynthesis and accumulation in maize kernels. Front. Plant Sci. 17, 1771678. doi: 10.3389/fpls.2026.1771678 41756656 PMC12932514

[B12] Herridge R. P. Day R. C. Baldwin S. Macknight R. C. (2011). Rapid analysis of seed size in Arabidopsis for mutant and QTL discovery. Plant Methods 7, 3. doi: 10.1186/1746-4811-7-3 21303553 PMC3046896

[B13] Hu R. Wang J. Yang H. Wei D. Tang Q. Yang Y. . (2023). Comparative transcriptome analysis reveals the involvement of an MYB transcriptional activator, SmMYB108, in anther dehiscence in eggplant. Front. Plant Sci. 14, 1164467. doi: 10.3389/fpls.2023.1164467 37521920 PMC10382176

[B14] Huang H. Gao H. Liu B. Qi T. Tong J. Xiao L. . (2017). Arabidopsis MYB24 regulates jasmonate-mediated stamen development. Front. Plant Sci. 8, 1525. doi: 10.3389/fpls.2017.01525 28928760 PMC5591944

[B15] Jiang L. Yue M. Liu Y. Zhang N. Lin Y. Zhang Y. . (2023). A novel R2R3-MYB transcription factor FaMYB5 positively regulates anthocyanin and proanthocyanidin biosynthesis in cultivated strawberries (Fragaria × ananassa). Plant Biotechnol. J. 21, 1140–1158. doi: 10.1111/pbi.14024 36752420 PMC10214752

[B16] Kuang L. Chen J. Bao X. Zhang D. Liu J. Wang W. . (2025). Environmental and phytohormonal factors regulating anthocyanin biosynthesis in fruits. Horticulturae 11, 681. doi: 10.3390/horticulturae11060681 30654563

[B17] Li C. Meng D. Piñeros M. A. Mao Y. Dandekar A. M. Cheng L. (2020). A sugar transporter takes up both hexose and sucrose for sorbitol-modulated *in vitro* pollen tube growth in apple. Plant Cell 32, 449–469. doi: 10.1105/tpc.19.00638 31826966 PMC7008483

[B18] Li C. Yu W. Xu J. Lu X. Liu Y. (2022). Anthocyanin biosynthesis induced by MYB transcription factors in plants. Int. J. Mol. Sci. 23, 11701. doi: 10.3390/ijms231911701 36233003 PMC9570290

[B19] Li J. Han G. Sun C. Sui N. (2019). Research advances of MYB transcription factors in plant stress resistance and breeding. Plant Signaling Behav. 14, 1613131. doi: 10.1080/15592324.2019.1613131 31084451 PMC6619938

[B20] Li Y. Gong K. Wang X. Sun Z. Ding F. (2025). Heat shock transcription factors as central integrators of plant stress responses: From thermotolerance to multi-stress resilience. Biology 14, 1800. doi: 10.3390/biology14121800 41463573 PMC12730419

[B21] Liu Y. Ma K. Qi Y. Lv G. Ren X. Liu Z. . (2021). Transcriptional regulation of anthocyanin synthesis by MYB-bHLH-WDR complexes in kiwifruit (Actinidia chinensis). J. Agric. Food. Chem. 69, 3677–3691. doi: 10.1021/acs.jafc.0c07037 33749265

[B22] Li Z. Lv S. Liu Y. Cao M. Zhang H. Hao Q. (2026). Transcriptome and metabolome analyses reveal the accumulation mechanism of carbohydrates during Paeonia ostii seed development. Biomolecules 16, 17. doi: 10.3390/biom16010017 41594559 PMC12838947

[B23] Mandaokar A. Browse J. (2009). MYB108 acts together with MYB24 to regulate jasmonate-mediated stamen maturation in Arabidopsis. Plant Physiol. 149, 851–862. doi: 10.1104/pp.108.132597 19091873 PMC2633834

[B24] Marciniak K. Przedniczek K. (2019). Comprehensive insight into gibberellin- and jasmonate-mediated stamen development 10, 811. doi: 10.3390/genes10100811 PMC6827089 31618967

[B25] Meng D. He M. Bai Y. Xu H. Dandekar A. M. Fei Z. . (2018). Decreased sorbitol synthesis leads to abnormal stamen development and reduced pollen tube growth via an MYB transcription factor, MdMYB39L, in apple (Malus domestica). New Phytol. 217, 641–656. doi: 10.1111/nph.14824 29027668

[B26] Meng J. Wang H. Chi R. Qiao Y. Wei J. Zhang Y. . (2023). The eTM–miR858–MYB62-like module regulates anthocyanin biosynthesis under low-nitrogen conditions in Malus spectabilis. New Phytol. 238, 2524–2544. doi: 10.1111/nph.18894 36942952

[B27] Muhammad N. Liu Z. Wang L. Yang M. Liu M. (2024). The underlying molecular mechanisms of hormonal regulation of fruit color in fruit-bearing plants. Plant Mol. Biol. 114, 104. doi: 10.1007/s11103-024-01494-1 39316226

[B28] Orozco-Arroyo G. Paolo D. Ezquer I. Colombo L. (2015). Networks controlling seed size in Arabidopsis. Plant Reprod. 28, 17–32. doi: 10.1007/s00497-015-0255-5 25656951

[B29] Rolletschek H. Muszynska A. Borisjuk L. (2021). The process of seed maturation is influenced by mechanical constraints. New Phytol. 229, 19–23. doi: 10.1111/nph.16815 32735708

[B30] Sharma H. Sharma P. Kumar A. Chawla N. Dhatt A. S. (2024). Multifaceted regulation of anthocyanin biosynthesis in plants: A comprehensive review. J. Plant Growth Regul. 43, 3048–3062. doi: 10.1007/s00344-024-11306-x 30311153

[B31] Shen X. Hu Z. Xiang X. Xu L. Cao J. (2019). Overexpression of a stamen-specific R2R3-MYB gene BcMF28 causes aberrant stamen development in transgenic Arabidopsis. Biochem. Biophys. Res. Commun. 518, 726–731. doi: 10.1016/j.bbrc.2019.08.119 31472956

[B32] Song S. Qi T. Huang H. Ren Q. Wu D. Chang C. . (2011). The jasmonate-ZIM domain proteins interact with the R2R3-MYB transcription factors MYB21 and MYB24 to affect jasmonate-regulated stamen development in Arabidopsis. Plant Cell 23, 1000–1013. doi: 10.1105/tpc.111.083089 21447791 PMC3082250

[B33] Su P. Sui C. Wang S. Liu X. Zhang G. Sun H. . (2023). Genome-wide evolutionary analysis of AUX/IAA gene family in wheat identifies a novel gene TaIAA15-1A regulating flowering time by interacting with ARF. Int. J. Biol. Macromol. 227, 285–296. doi: 10.1016/j.ijbiomac.2022.12.175 36549029

[B34] Sui C. Cheng S. Wang D. Lv L. Meng H. Du M. . (2023). Systematic identification and characterization of the soybean (Glycine max) B-box transcription factor family. Biotechnol. Biotechnol. Equip. 37, 104–116. doi: 10.1080/13102818.2022.2155570 37339054

[B35] Sun B. Zhu Z. Chen C. Chen G. Cao B. Chen C. . (2019). Jasmonate-inducible R2R3-MYB transcription factor regulates capsaicinoid biosynthesis and stamen development in Capsicum. J. Agric. Food. Chem. 67, 10891–10903. doi: 10.1021/acs.jafc.9b04978 31505929

[B36] Takos A. M. Jaffeé F. W. Jacob S. R. Bogs J. Robinson S. P. Walker A. R. (2006). Light-induced expression of a MYB gene regulates anthocyanin biosynthesis in red apples. Plant Physiol. 142, 1216–1232. doi: 10.1104/pp.106.088104 17012405 PMC1630764

[B37] Vergès V. Dutilleul C. Godin B. Collet B. Lecureuil A. Rajjou L. . (2021). Protein farnesylation takes part in Arabidopsis seed development. Front. Plant Sci. 12, 620325. doi: 10.3389/fpls.2021.620325 33584774 PMC7876099

[B38] Wang D. Du M. Li J. Yin S. Sun Y. Guo S. (2023). Overexpression of GhSWEET42, a SWEET-like gene from cotton, enhances the oil content and seed size. Biotechnol. Biotechnol. Equip. 37, 2266529. doi: 10.1080/13102818.2023.2266529 37339054

[B39] Wang M. Zhang S. Ding F. (2020). Melatonin mitigates chilling-induced oxidative stress and photosynthesis inhibition in tomato plants. Antioxidants 9, 218. doi: 10.3390/antiox9030218. [Online]. 32155702 PMC7139585

[B40] Wang T. Jin Y. Deng L. Li F. Wang Z. Zhu Y. . (2024). The transcription factor MYB110 regulates plant height, lodging resistance, and grain yield in rice. Plant Cell 36, 298–323. doi: 10.1093/plcell/koad268 37847093 PMC10827323

[B41] Wang X. Niu Y. Zheng Y. (2021). Multiple functions of MYB transcription factors in abiotic stress responses. Int. J. Mol. Sci. 22, 6125. doi: 10.3390/ijms22116125 34200125 PMC8201141

[B42] Wang Y. Sun J. Wang N. Xu H. Qu C. Jiang S. . (2018). MdMYBL2 helps regulate cytokinin-induced anthocyanin biosynthesis in red-fleshed apple (Malus sieversii f. niedzwetzkyana) callus. Funct. Plant Biol. 46, 187–196. doi: 10.1071/FP17216 32172760

[B43] Wiese A. J. Torutaeva E. Honys D. (2024). The transcription factors and pathways underpinning male reproductive development in Arabidopsis. Front Plant Sci. 15, 1354418. doi: 10.3389/fpls.2024.1354418 38390292 PMC10882072

[B44] Wilson Z. A. Song J. Taylor B. Yang C. (2011). The final split: the regulation of anther dehiscence. J. Exp. Bot. 62, 1633–1649. doi: 10.1093/jxb/err014 21325605

[B45] Wu B. Li R. Ye M. Wang W. Zhang H. Zhuang K. . (2025). Exogenous melatonin enhances the cold resistance of pepper (Capsicum annuum L. var. cerasiforme Bailey) by promoting the synthesis of long-chain alkanes in the epicuticular wax. J. Pineal Res. 77, e70097. doi: 10.1111/jpi.70097 41267367

[B46] Wu X. Xia M. Su P. Zhang Y. Tu L. Zhao H. . (2024). MYB transcription factors in plants: A comprehensive review of their discovery, structure, classification, functional diversity and regulatory mechanism. Int. J. Biol. Macromol. 282, 136652. doi: 10.1016/j.ijbiomac.2024.136652 39427786

[B47] Yang K. An J. P. Li C. Y. Shen X. N. Liu Y. J. Wang D. R. . (2021a). The apple C2H2-type zinc finger transcription factor MdZAT10 positively regulates JA-induced leaf senescence by interacting with MdBT2. Hortic. Res. 8, 159. doi: 10.1038/s41438-021-00593-0 34193837 PMC8245655

[B48] Yang J. Chen Y. Xiao Z. Shen H. Li Y. Wang Y. (2022). Multilevel regulation of anthocyanin-promoting R2R3-MYB transcription factors in plants. Front. Plant Sci. 13, 1008829. doi: 10.3389/fpls.2022.1008829 36147236 PMC9485867

[B49] Yang K. Li C.-Y. An J.-P. Wang D.-R. Wang X. Wang C.-K. . (2021b). The C2H2-type zinc finger transcription factor MdZAT10 negatively regulates drought tolerance in apple. Plant Physiol. Biochem. 167, 390–399. doi: 10.1016/j.plaphy.2021.08.014 34404010

[B50] Yang C. Xu Z. Song J. Conner K. Vizcay Barrena G. Wilson Z. A. (2007). Arabidopsis MYB26/MALE STERILE35 regulates secondary thickening in the endothecium and is essential for anther dehiscence. Plant Cell 19, 534–548. doi: 10.1105/tpc.106.046391 17329564 PMC1867336

[B51] Zhang J. Zhang X. Liu X. Pai Q. Wang Y. Wu X. (2023). Molecular network for regulation of seed size in plants. Int. J. Mol. Sci. 24, 10666. doi: 10.3390/ijms241310666 37445843 PMC10341960

[B52] Zhang M. Zhao J. Wang N. Hu D.-G. Borejsza-Wysocka E. Cheng L. (2026). Overexpression of a tonoplast malate transporter gene leads to enhanced anthocyanin biosynthesis in apple. Plant Biotechnol. J. 24, 620–638. doi: 10.1111/pbi.70368 40958366 PMC12906801

[B53] Zhang D. Zhou H. Zhang Y. Zhao Y. Zhang Y. Feng X. . (2025). Diverse roles of MYB transcription factors in plants. J. Integr. Plant Biol. 67, 539–562. doi: 10.1111/jipb.13869 40013511

[B54] Zhao K. Zhang Q. Wang Y. Wei Q. Wang Y. (2025). Advances in the biosynthesis and regulatory mechanisms of anthocyanins in horticultural plants: a comprehensive review. Trop. Plants 4, e010. doi: 10.48130/tp-0025-0002

[B55] Zúñiga-Mayo V. M. Durán-Medina Y. Marsch-Martínez N. de Folter S. (2023). “ Hormones and flower development in Arabidopsis,” in Flower Development : Methods and Protocols. Eds. Riechmann J. L. Ferrándiz C. ( Springer US, New York, NY), 111–127. 10.1007/978-1-0716-3299-4_5 37540356

