# Supplementary material for: MdMYB41L mediates stamen development and anthocyanin biosynthesis in apple
Source: Front Plant Sci. 2026 Jul 30;17:1905435. doi: 10.3389/fpls.2026.1905435 (PMC13468913; doi:10.3389/fpls.2026.1905435)
Supplement: Supplementary file 1 [file Table1.pdf]

Supplementary data

Supplementary Table 1.
Primers used in this study

Primer name	sequence (5' to 3')
MdMYB41L-PRI-F	GGATCCATGGTTAGAGCCCCTTGTTG
MdMYB41L-PRI-R	GTCGACATCTGCAAGAACATACTCTAGAA
MdMYB41L(qRT)-F	GATGGCTTCCACGCGATTTC
MdMYB41L(qRT)-R	TGCAGGAAGTGGGTGGTTTT
AtACTIN-F	TTTGGAGCCTGGGACTATGGAT
AtACTIN-R	ACGGGGGAATGGGATGAGAT
MdACTIN-F	TGACCGAATGAGCAAGGAAATTACT
MdACTIN-R	TACTCAGCTTTGGCAATCCACATC
